# Physicomechanical and Antimicrobial Characteristics of Cement Composites with Selected Nano-Sized Oxides and Binary Oxide Systems

**DOI:** 10.3390/ma15020661

**Published:** 2022-01-16

**Authors:** Patryk Jędrzejczak, Łukasz Ławniczak, Agnieszka Ślosarczyk, Łukasz Klapiszewski

**Affiliations:** 1 Institute of Chemical Technology and Engineering, Faculty of Chemical Technology, Poznan University of Technology, Berdychowo 4, PL-60965 Poznan, Poland; patryk.jedrzejczak@doctorate.put.poznan.pl (P.J.); lukasz.lawniczak@put.poznan.pl (Ł.Ł.); 2 Institute of Building Engineering, Faculty of Civil and Transport Engineering, Poznan University of Technology, Piotrowo 3, PL-60965 Poznan, Poland; agnieszka.slosarczyk@put.poznan.pl

**Keywords:** oxides, binary oxides, cement mortar, physicomechanical characteristics, antimicrobial properties

## Abstract

In recent years, increasing attention has been paid to the durability of building materials, including those based on cementitious binders. Important aspects of durability include the increase of the strength of the cement matrix and enhancement of material resistance to external factors. The use of nanoadditives may be a way to meet these expectations. In the present study, zinc, titanium and copper oxides, used in single and binary systems (to better the effect of their performance), were applied as additives in cement mortars. In the first part of this work, an extensive physicochemical analysis of oxides was carried out, and in the second, their application ranges in cement mortars were determined. The subsequent analyses were employed in determining the physicochemical properties of pristine oxides: Fourier transform infrared spectroscopy (FTIR), energy dispersive X-ray fluorescence (EDXRF), scanning electron microscopy (SEM), measurement of the particle size distribution, as well as zeta potential measurement depending on the pH values. Influence on selected physicomechanical parameters of the cement matrix and resistance to the action of selected Gram-positive and Gram-negative bacteria and fungi were also examined. Our work indicated that all nanoadditives worsened the mechanical parameters of mortars during the first 3 days of hardening, while after 28 days, an improvement was achieved for zinc and titanium(IV) oxides. Binary systems and copper(II) oxide deteriorated in strength parameters throughout the test period. In contrast, copper(II) oxide showed the best antibacterial activity among all the tested oxide systems. Based on the inhibitory effect of the studied compounds, the following order of microbial susceptibility to inhibition of growth on cement mortars was established (from the most susceptible, to the most resistant): *E. coli* < *S. aureus* < *C. albicans* < *B. cereus* = *P. aeruginosa* < *P. putida*.

## 1. Introduction

In the 21st century, increasing emphasis is being placed on sustainable construction. This is because cement production is a sector that directly and indirectly contributes to the emission of a huge amount of greenhouse gases and also requires extensive energy input [1]. Thus, civilizational and economic development drives the need to search for cement composites with improved properties. A promising and currently widely developed solution to these two issues is the use of nanotechnology and nanomaterials. An important publication regarding the application of nanotechnology and nanomaterials in construction, especially in concretes, is the pioneering work of Sanchez and Sobolev [2], which recognized the great potential of using nanotechnology to enhance the properties of concrete and to create innovative, functional cement-based composites with exceptional mechanical, thermal and electrical properties [2]. Therefore, in the field of construction and building materials, research should be continued into the application of nanotechnology [3].

Currently, silica is one of the most widely used nanomaterials in construction. The extensive application of nano-SiO_2_ results from its nanometric size, highly developed BET (Brunauer-Emmett-Teller) surface area and pozzolanic activity [4]. Nanosilica is used to modify various cement and polymer composites in order to bolster their properties, both in plastic and hardened states. The introduction of nanometric SiO_2_ improves their strength, flexibility, durability and workability. For this reason, SiO_2_ nanoparticles are used as additives, e.g., for high-performance and self-compacting concretes [5]. Additionally, nano-SiO_2_ improves the compressive strength of cement composites. The enhancement is proportional to the development of its specific surface. This is due to the fact that such silica includes more Si-OH groups on its surface, which contribute to greater reactivity [4]. Herein, nanosilica contributes to the consumption of Ca(OH)_2_ and the formation of “additional” C-S-H by participating in pozzolanic reactions. Nanomaterials, including nanometric SiO_2_, also act as centers of crystallization of cement hydrates, due to which the cement hydration process is accelerated. As a result of the above, a material with a more compact structure, reduced porosity, improved strength and increased resistance to external factors is obtained [5]. Beyond the aforementioned, He et al. found that nanosilica affects the content and distribution of the pore size in hardened air-entrained concrete [6]. Moreover, nano-SiO_2_ introduced into concretes containing steel reinforcements increases their corrosion resistance [7].

Aluminum oxide [8,9,10,11], titanium(IV) oxide [11,12] and iron(III) oxide are other materials that are now being introduced into cement composites [11,13]. Carbon nanomaterials are also beginning to be applied in building material technology. Carbon nanotubes [14,15,16], carbon nanofibers [16] and graphene oxide [17] are among the most frequently studied materials of this type in terms of their use as additives to cement composites. The introduction of the mentioned materials clearly affects the properties of cement composites, slows down the formation and development of cracks, and contributes to the improvement of mechanical properties, reduction of porosity and the obtaining of composites of higher density. Furthermore, additives in the form of carbon nanotubes or carbon nanofibers contribute to improving electrical conductivity properties [16]. Of note, the article by Yoo et al. demonstrates that multi-wall carbon nanotubes have the most influence on the self-sensing capacity of cement mortar compared to graphite nanofibers and graphene [18].

Among the materials mentioned above, titanium(IV) oxide deserves special attention, as it exhibits the ability to photocatalyze chemical reactions and is also characterized by low cost, chemical stability and low toxicity [19]. In the case of TiO_2_, attempts are currently being made to incorporate it into building materials in order to grant them the ability to degrade pollutants. In the article by Kim et al., two different titanium(IV) oxides were introduced into cement composites, namely: (i) rutile titanium dioxide produced by commercial annealing P25 and (ii) R996, which is commercially available TiO_2_ coated with Al_2_O_3_ and ZrO_2_. The first of the cited materials is characterized by higher photocatalytic activity. In the course of the research, it was observed that the resulting materials exhibited comparable photocatalytic activity induced by visible light towards NO oxidation [20].

Titanium(IV) oxide, when used as a cement admixture, increases the consistency of fresh concrete mixture, reduces water absorption and improves compressive strength. What is more, in the case of a cement composite modified with styrene-butadiene-rubber latex in the form of a powder, the addition of TiO_2_ in the amount of 2% by weight contributes to a reduction of consistency, a significant increase in compressive strength and almost does not affect water adsorption compared to samples not containing the said oxide [21]. 

In research, the introduction of TiO_2_-SiO_2_ nanocomposites into a cement matrix has been found to improve its compressive strength. This effect is due to the good pozzolanic activity of silica and the development of a denser structure. The presence of TiO_2_ in this oxide system contributes to the self-cleaning properties of the resulting material in both UV and visible light [22]. 

Titanium(IV) oxide is also used to make building materials that protect against gamma radiation. For example, in the work by Dezhampanah and others, concrete reinforced with polypropylene fibers was produced that contained magnetite aggregate and TiO_2_ in an amount of 0–8% by weight, due to which it was characterized by shielding properties for the above-mentioned radiation [23]. Additionally, the work by Nikbin et al. reported that the increase in the content of titanium(IV) oxide (0–8% by weight) in heavy concrete containing magnetite aggregate contributes to the improvement of its ability to protect against gamma radiation [24].

The formation of biofilms, which consist of various microorganisms, including. microalgae, fungi or lichens, on structures made of cement composites is a major problem that contributes to the deterioration of their aesthetics and may be responsible for reducing their durability [25,26]. For this purpose, materials with properties that counteract this phenomenon are sought. Activation of antibacterial properties in building materials by introduction of appropriate admixtures is also important whenever the presence of microorganisms is not desired, i.e., in medical facilities and food processing plants [25,27,28]. 

In order to obtain building materials with antimicrobial properties, zinc oxide or copper(II) oxide (among others) are introduced into them. For example, in work by Vavouraki et al., nanometric ZnO and CuO were introduced into an inorganic polymer based on ground waste concrete (this is considered to be a “third generation” concrete). The addition of the aforementioned oxides increased the compressive strength and gave the material antimicrobial properties against *E. coli* [29]. Furthermore, in an article by Klapiszewska et al., it was observed that cement composites containing pristine zinc oxide or a zinc oxide-lignin hybrid material exhibited antibacterial and antifungal properties. These properties were slightly weakened as a result of combining zinc oxide with lignin in the hybrid material [30].

The literature also contains information about attempts to simultaneously introduce more than one oxide into the cement composite. For example, the publication by Oltulu et al. investigated the effect of adding single silicon(IV), aluminum and iron(III) oxides, as well as two- and three-component systems. The ratios of the introduced pristine substances and their mixtures amounted to 0.5, 1.25 and 2.5% of the whole by weight in relation to the amount of binder. The use of all oxides, both separately and in two- and three-component systems (with the exception of nano-SiO_2_ in the amount of 2.5% by weight), contributed to the improvement of the 28-day compressive strength of the obtained cement composite. Nevertheless, these two- and three-component systems did not allow obtaining mortars with better values of compressive strength and capillary permeability than in the case of materials containing single oxides [31]. 

In another work by the Oltulu team, fly ash was incorporated as a mineral additive instead of the previously employed silica dust. The obtained results differed from those obtained in the previous work, as the best physicomechanical properties were observed in the case of the cement composite containing the three-component system consisting of nano-SiO_2_, nano-Al_2_O_3_ and nano-Fe_2_O_3_, which constituted 1.25% of the whole by weight [32].

To the best of the authors’ knowledge, there are no studies in the literature regarding the mechanical and antibacterial properties of cement composites containing admixtures in the form of more than one oxide, i.e., zinc oxide and copper(II) oxide, as well as titanium(IV) oxide and copper(II) oxide. Therefore, the aim of this study was to determine and compare the effect of zinc oxide, copper(II) oxide and titanium(IV) oxide on the properties of the obtained cement composites, using them individually and in systems consisting of two oxides. It should be noted that in the research, zinc oxide and titanium(IV) oxide were not added to any of the samples simultaneously. 

In the first part of the work, the characteristics of pristine oxides were conducted using analyses such as: Fourier transform infrared spectroscopy (FTIR), energy dispersive X-ray fluorescence (EDXRF) and scanning electron microscopy (SEM). In this research, particle size distribution was measured, and zeta potential curves were plotted relative to pH values. 

These oxides were then used as admixtures for cement composites, which were subsequently characterized. For this purpose, the consistency of fresh cement mortar, the density, as well as the compressive and flexural strength after 3 and 28 days of hardening were measured. 

To discern the antibacterial properties, both the pristine oxides and the produced cement composites were subjected to appropriate tests. In order to properly evaluate the antimicrobial effects of inorganic oxides, it was important to incorporate various microorganisms into the study. For this reason, the tests included blending into the composites commonly occurring opportunistic pathogens such as Gram-positive bacteria (e.g., *B. cereus*—responsible for foodborne diarrhoea syndromes, and *S. aureus*—associated with skin infections), Gram-negative bacteria (e.g., *E. coli* or *P. aeruginosa*—pathogens of the intestinal and respiratory tracts, respectively), as well as fungi (e.g., *C. albicans*—a common cause of candidiasis), as these species are most commonly present in human habitats.

## 2. Materials and Methods

### 2.1. Materials

The following substances were employed in this study: (i) zinc oxide—ZnO (CAS number: 1314-13-2), (ii) copper(II) oxide—CuO (CAS number: 1317-38-0) and (iii) titanium(IV) oxide—TiO_2_ (CAS number: 13463-67-7), which were supplied by Sigma-Aldrich (Steinheim am Albuch, Germany). Portland CEM I 42.5R cement from Górażdże Cement SA, Górażdże, Poland, which meets the requirements of PN-EN 197-1, was used to prepare the cement mortars. Standard quartz sand (standard requirements according to PN-EN 196-1) from Kwarcmix (Tomaszów Mazowiecki, Poland) was incorporated as an aggregate.

### 2.2. Physicochemical and Dispersive-Microstructure Characteristics

#### 2.2.1. Fourier Transform Infrared Spectroscopy (FTIR)

Fourier transform infrared spectroscopy was used to identify characteristic bands derived from the functional groups present in the structures of pristine components (ZnO, CuO and TiO_2_). In order to obtain FTIR spectra, a Vertex 70 spectrometer (Bruker GmbH, Mannheim, Germany) was used that was equipped with a sensitive MCT (mercury cadmium telluride) detector. It should be noted that this apparatus provides a very high scanning resolution of the samples, which exceeds 0.5 cm^−1^. FTIR analysis was performed in the wavenumber range of 4000–450 cm^−1^, and the number of scans was equal to 64. KBr pellets were prepared to perform the FTIR analysis of the used oxides. Their preparation consisted of mixing 250 mg of anhydrous KBr and approx. 1 mg of the analyzed substance, and then exposing this mixture to the pressure of 10 MPa for 10 min.

#### 2.2.2. Energy Dispersive X-ray Fluorescence (EDXRF)

The surface composition of the analyzed oxides was determined using an Epsilon4 EDXRF spectrometer (PANalytical, Malvern, UK), operating based on energy dispersion (EDXRF). The spectrometer was equipped with an X-ray tube with an Ag 5W anode and a 50 kV generator with 500 uA current, a set of six measurement filters and a high-resolution SDD semiconductor detector cooled by the Peltier effect.

#### 2.2.3. Scanning Electron Microscopy (SEM) and Particle Size Analysis

Microstructural analysis of the obtained cement composites was carried out by means of a TESCAN3VEGA scanning electron microscope (Tescan, Brno, Czech Republic). In order to compare and determine the dispersion properties of the used oxides, the samples were analyzed for particle size distribution utilizing a Zetasizer Nano ZS device (Malvern Instruments Ltd., Malvern, UK). The apparatus employs the non-invasive back scatter technology (NIBS), which enables the examination of particle sizes in the range of 0.6–6000 nm.

#### 2.2.4. Porous Structure Properties

An ASAP 2020 physical sorption analyzer (Micrometrics Instrument Co., Norcross, GA, USA) was applied to determine the properties of the porous structure of the tested oxides. Prior to the analysis, the samples were degassed for 4 h at 120 °C. The surface area was determined by the multipoint BET method (Brunauer–Emmett–Teller) using adsorption data for the relative pressure (p/p_0_) ranging from 0.05–0.30. The pore size distribution was ascertained by means of the desorption isotherm and based on the BJH model (Barrett–Joyner–Halenda). Due to the use of this highly accurate apparatus, it was possible to determine the BET surface area, total pore volume and average pore size with an accuracy of 0.1 m^2^/g, 0.001 cm^3^/g and 0.01 nm, respectively.

#### 2.2.5. Zeta Potential Analysis

The Zetasizer Nano ZS apparatus was employed to measure the electrophoretic mobility of the tested oxides based on laser Doppler velocity (LDV). By means of the software of the Zetasizer Nano ZS analyzer, the electrokinetic potentials of the oxides were indirectly determined by calculating them on the basis of their electrophoretic mobility value by applying the Henry equation. The zeta potential was determined over the entire pH range in the presence of an electrolyte, which in this case was 0.001 M NaCl solution, and this allowed for the determination of electrokinetic curves. The mean standard errors of the measurement of the determined zeta potential and the pH measurement were ±2 mV and ±0.1, respectively.

### 2.3. Preparation of Cement Composites

In the second part of the work, samples containing various weight fractions of zinc oxide, copper(II) oxide and titanium(IV) oxide were prepared in order to determine the influence of the aforementioned oxides on the properties of cement composites. The following ingredients were used to prepare the beams: 450 g of Portland CEM I 42.5R cement, 225 mL of distilled water, 1350 g of aggregate in the form of standard quartz sand and the appropriate amount of oxide or binary oxide system. The ratios of ZnO, CuO and TiO_2_ in relation to the cement mass differed depending on the sample. The introduced admixture masses are summarized in Table 1.

The cement was placed directly in the mixing tank, while the oxides were pre-dispersed in 50 mL of distilled water by means of a magnetic stirrer, forming a suspension, and were dosed into the tank only in this form. The ingredients prepared in this way were then mixed according to the standard procedure of EN 196-1. In the next step, the aggregate was added gradually over 30 s by means of an automatic mixer feeder, starting the addition process after the first 30 s of mixing had passed and using a low agitator speed. The stirrer speed was then increased for another 30 s. The mixing process was subsequently stopped for 60 s to allow the mortar to be picked up from the walls and transferred to the center of the tank. High-speed agitation was then started and mixing was carried out for 60 s. In the next step, part of the mix was taken, returned to the system and combined with the rest of the mortar to perform the flow test. Eventually, all the mortar was poured into the beam molds. The mortar was placed in two layers and each was compacted with a compactor, applying 60 strokes per layer. The top surfaces were then flattened before resting the mold. The 40 mm × 40 mm × 160 mm beams were separated approximately 24 h later and placed in water, where they remained until the mechanical and antibacterial properties were measured.

### 2.4. Characterization of Cement Composites

#### 2.4.1. Determination of the Consistency of Fresh Cement Mortar

In order to determine the consistency of fresh cement mortar, a vibrating table, a compactor, a truncated cone-shaped form and a steel ruler were used. The table diameter was equal to 300 mm, the lifting height was 10 mm, and the conical form was 60/100 mm in diameter and 70 mm in height. The measurement was carried out in accordance with the EN 1015-3 standard. To obtain the consistency value of fresh cement mortar, two perpendicular diameters of the sample flows were measured twice, and the results obtained in this way were averaged.

#### 2.4.2. Determination of the Density

The density was assessed as follows: the beams made of the analyzed cement mortars were first pulled out of the water, and then their weight was measured. For each sample, 3 selected beams were weighed, after which the obtained results were averaged and divided by the volume of the beam (dimensions of 40 mm × 40 mm × 160 mm, i.e., 256 cm^3^), thus obtaining the density of individual samples.

#### 2.4.3. Compressive and Flexural Strength Tests

Both the determination of the compressive strength and the bending strength were carried out according to the procedure described in the EN 196-1 standard. To perform the measurements, a Servo-Plus Evolution apparatus (MATEST S.p.A., Treviolo, Italy) was employed. Before the assessment of the above-mentioned parameters, the beams were immersed in water and were pulled out of it for the time of the measurement. In order to measure the flexural strength, samples with a square cross-section (40 mm × 40 mm × 160 mm) were placed axially on two supporting rollers, spaced 100 mm apart. A load was then applied by means of a load roller and the set force was gradually increased by 50 ± 10 N/s until the beam broke. The measurement of the flexural strength was repeated on three beams, and the obtained results were then averaged.

The beam halves obtained from the measurement of the flexural strength were then used to determine the compressive strength. For this purpose, samples of cement composites were placed between two clamping plates with a square cross-section and the load was gradually increased by 2.4 ± 0.2 kN/s until the sample was crushed. In order to ascertain the compressive strength of the tested cement composites, all 6 beam halves were used, and the obtained results were averaged.

The compressive and flexural strengths were measured on the samples after 3 and 28 days of hardening.

### 2.5. Assessment of Antibacterial Properties

Assessment of antibacterial properties was carried out based on a two-step analysis: at first, the microbial purity of solid plates was evaluated, followed by a determination of antimicrobial effect of powdered materials expressed as MIC (minimal inhibitory concentration) or MBC/MFC (minimal bactericidal/fungicidal concentration) as described in [30].

Microbial purity was evaluated by placing 5 mm × 5 mm × 2 mm plates of the studied materials on Petri dishes filled with sterile tryptic soy agar. The dishes were closed and incubated at 30 °C for 24 h. Afterwards, the microbial growth was established based on a visual analysis. All samples were prepared in triplicate.

In turn, determination of antimicrobial activity for the powdered admixtures was carried out against selected species of Gram-negative bacteria (*Pseudomonas putida*, *Pseudomonas aeruginosa*, *Escherichia coli*), Gram-positive bacteria (*Bacillus cereus*, *Staphylococcus aureus*) and a single fungus (*Candida albicans*). Each of the tested compounds (100 mg) was prepared in a 2 mL Eppendorf vial and sterile water was added. The samples were then shaken using a mini-shaker and a homogeneous suspension was obtained. Next, a series of dilutions were carried out in order to obtain half of the previous concentration each time, which resulted in 8 suspensions. All solutions were stored in a refrigerator at 4 °C for no longer than a week.

Cultures of each species were transferred from agar plates into 20 mL 50% TSB broth (Sigma-Aldrich, Saint Louis, MO, USA). Afterwards, each of the cultures was incubated for 24 h at 30 °C. When the cell suspension reached optical density equal to approx. OD600 = 0.1 (which corresponds to 10^6^ cells per millilitre), the biomass was diluted (1:50) to give 2 × 104 cells per millilitre.

The antimicrobial activity test was carried out in accordance with the European Committee on Antimicrobial Susceptibility Testing, using the microdilution method according to the EUCAST guidelines [33,34]. In a sterile 96-well plate, 50 μL suspensions of each dilution were placed from the highest to the lowest dilution, starting from the first row of the plate. Subsequently, 200 μL of microorganisms suspension (2 × 10^4^ CFU per mL) with resazurin (4 mL of dilution at 0.5 mg/mL added to 20 mL of microbial suspension) was introduced. The concentrations ranged from 0.01 to 20 g/L. Microorganisms with resazurin but without the analyzed compounds (biotic control), as well as compound solutions with resazurin lacking microorganisms (abiotic control) were used as controls.

Three replications were prepared for each of the tested compounds. Plates were then incubated at 30 °C for 24 h, with constant stirring on a rocker shaker, after which the results of MIC and MBC or MFC were determined.

## 3. Results and Discussion

### 3.1. Physicochemical and Dispersive-Microstructure Characteristics of Pristine Oxides

#### 3.1.1. FTIR Spectroscopy

**Figure 1:**
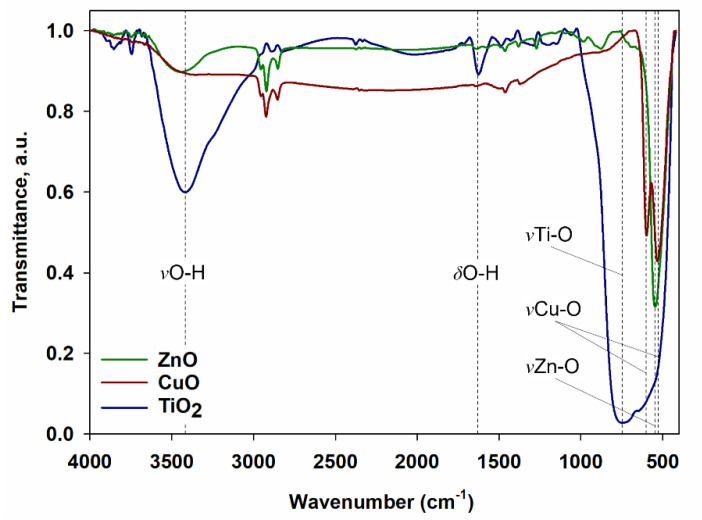
FTIR spectra of zinc oxide, copper(II) oxide and titanium(IV) oxide.

Fourier transform infrared spectroscopy was applied to confirm the characteristic functional groups of the used oxides. As a result of the analysis, spectra of pristine oxides were obtained (see Figure 1). In the case of the FTIR spectrum of titanium(IV) oxide, three bands are clearly visible. The bands with the maxima at the wavenumber of 3414 cm^−1^ and 1624 cm^−1^ originate from the stretching and bending vibrations of the -OH groups present on the surface of TiO_2_ particles, while the broad band that can be observed in the range 1000–400 cm^−1^ is attributed to vibrational stretching of the Ti-O-Ti groups. In the publication of Jo and Natarajan, which also analyzed TiO_2_ by means of Fourier transform infrared spectroscopy, bands at similar wavenumber values were observed [35]. In the publication of León et al., the occurrence of three bands was also observed from the stretching and bending vibrations of the -OH groups and from the Ti-O-Ti stretching vibrations [36].

In the case of the analysis of the spectrum of copper(II) oxide, two bands can be observed at the wavenumber in the range of 600–500 cm^−1^, which are attributed to the monoclinic phase of CuO nanostructures [37,38]. The bands with maxima at 596 cm^−1^ and 531 cm^−1^ correspond to the stretching vibrations of the Cu-O groups [39]. Based on the analysis of the FTIR spectrum, the presence of another phase of this oxide, namely, Cu_2_O, was excluded. The exclusion was made on the basis of the absence of an additional band in the range of the wavenumber 660–605 cm^−1^ [37,39].

When analyzing the spectrum of zinc oxide, a wide band with a maximum at the wavenumber in the range of 3600–3300 cm^−1^ can be observed, which is characteristic for the stretching vibrations of the hydroxyl groups. The narrow band that can be observed at the wavenumber of 544 cm^−1^ originates from the stretching vibrations of the Zn-O groups. In the work of Klapiszewska et al., an additional band in the ZnO spectrum was noticed with a maximum at the wavenumber of 1610 cm^−1^, which was attributed to water physically adsorbed on the oxide surface [30]. The performed observations and the assigned band at the wavenumber equal to 544 cm^−1^ are in accordance with the literature data [40,41,42].

In the case of the FTIR spectra of zinc oxide and copper(II) oxide, there are visible bands with maxima at the wavenumbers of 2955, 2920 and 2851 cm^−1^. These are characteristic of the stretching vibrations of C-H bonds, present e.g., in -CH_3_ or -CH_2_ groups. The appearance of these groups may be the result of some slight impurities left over from the manufacturing process.

#### 3.1.2. Energy Dispersive X-ray Fluorescence

**Figure 2:**
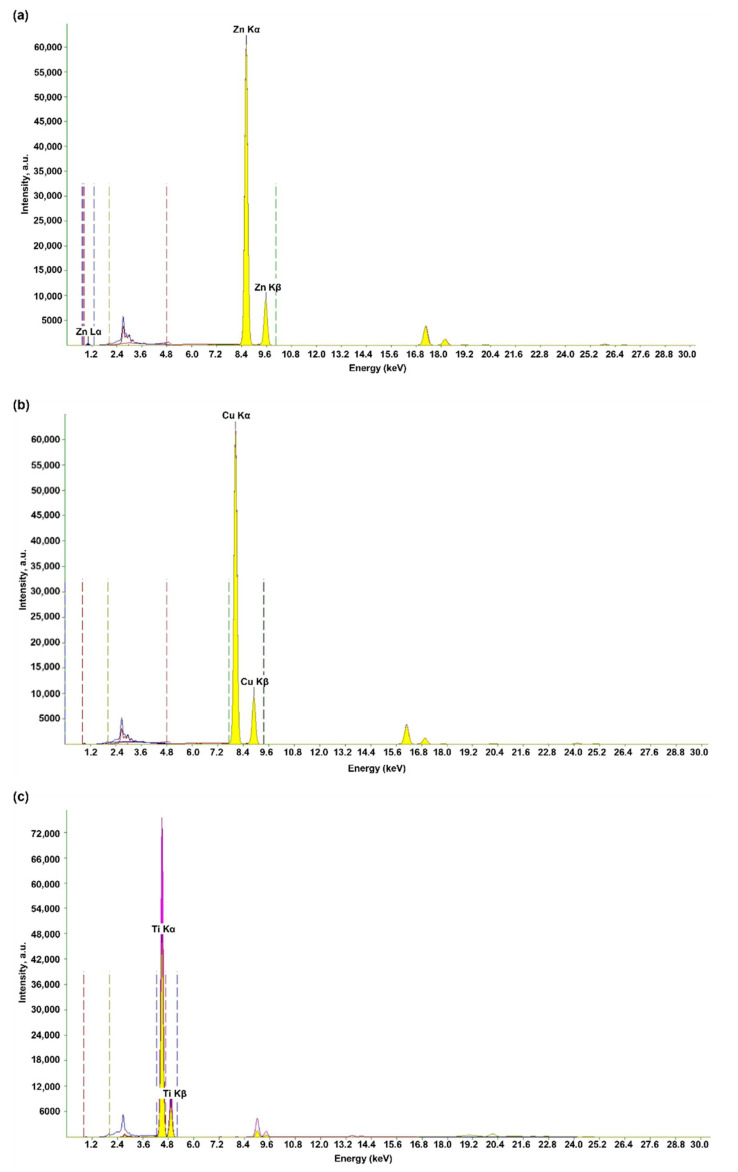
The obtained EDXRF spectra for the analyzed oxides: zinc oxide (**a**); copper(II) oxide (**b**); titanium(IV) oxide (**c**).

Energy dispersive X-ray fluorescence is another research technique that was applied to confirm the composition of oxides. Due to the conducted measurements, it was possible to conclude that the analyzed oxides, i.e., ZnO, CuO and TiO_2_, consist mainly of these oxides. The obtained EDXRF spectra for the tested oxides are shown in Figure 2. 

In the case of zinc oxide, characteristic peaks of Zn L_α_, Zn K_α_ and Zn K_β_ can be noticed, while the CuO spectrum shows the Cu K_α_ and Cu K_β_ peaks that indicate copper(II) oxide. Titanium(IV) oxide was also confirmed by analyzing its EDXRF spectrum, as the characteristic peaks of Ti K_α_ and Ti K_β_ were observed. The obtained peaks for the tested oxides are consistent with the literature data [43,44,45,46,47,48].

#### 3.1.3. Scanning Electron Microscopy and Particle Size Analysis

Another important part of this work was the analysis of the dispersion and morphological properties of the tested oxides. For this purpose, particle size distributions were measured, polydispersity indices (PdI) were determined, and SEM micrographs were analyzed. The data obtained from the measurement of the particle size distribution and the polydispersity index are summarized in Table 2. Upon analyzing the presented sizes, it can be seen that titanium(IV) oxide is the least homogeneous in terms of particle size among the tested oxides, as its PdI is equal to 0.788. Zinc oxide and copper (II) oxide, in contrast, possess similar PdI, which are respectively equal to 0.139 and 0.140. As a result of this, it can be concluded that these oxides are more homogeneous in terms of particle size compared to TiO_2_. 

**Figure 3:**
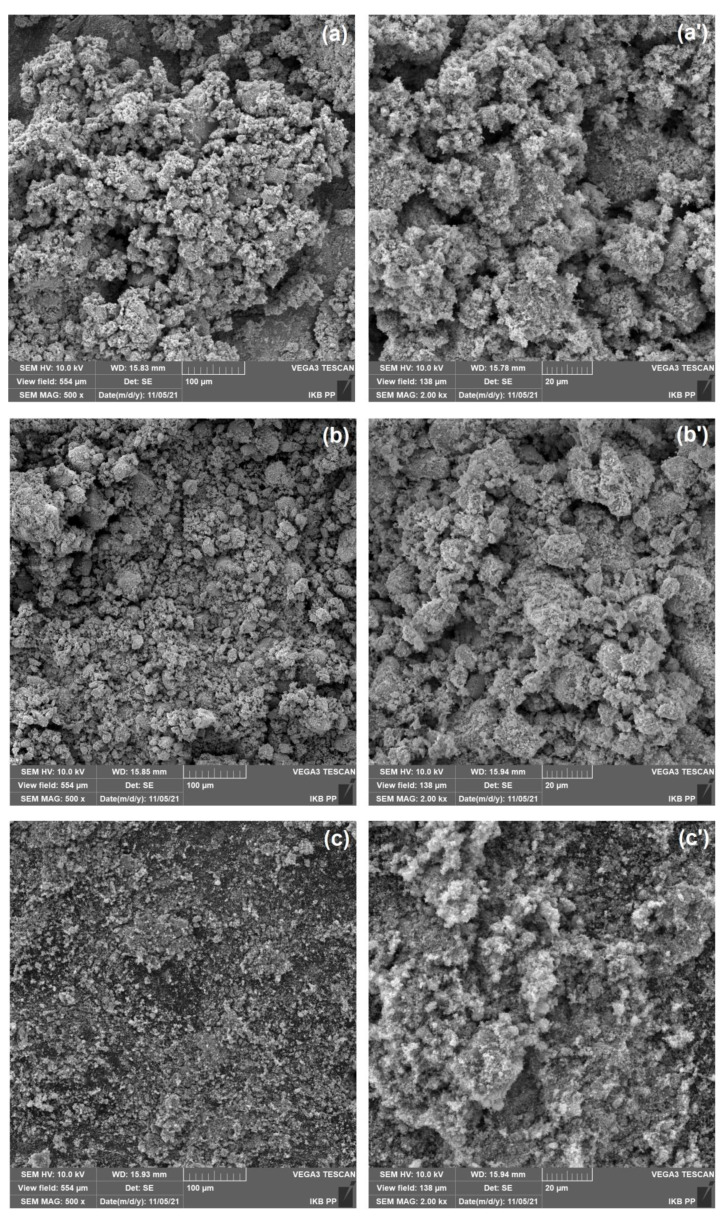
SEM images of ZnO (**a**,**a’**), CuO (**b**,**b’**) and TiO_2_ (**c**,**c’**) at two different magnifications.

The sizes of titanium(IV) oxide particles ranged from 190 to 396 nm. Zinc oxide and copper(II) oxide exhibited larger particle sizes, which ranged from 142 to 712 nm and from 220 to 1484 nm, respectively. Micrographs obtained by scanning electron microscopy (see Figure 3) correlate very well with the results of the previously described particle size measurement and polydispersity index. Moreover, SEM images analysis shows that all tested oxides tend to form aggregates (structures with a size of up to 1 μm) and agglomerates (structures with a size greater than 1 μm).

#### 3.1.4. Porous Structure Properties

The determination of the properties of the porous structure of zinc oxide, copper(II) oxide and titanium(IV) oxide is of great importance in terms of their potential use as admixtures for cement composites. In order to characterize the porous structure, the following parameters were determined: BET surface area, total volume of pores and average size of pores. The obtained data are summarized in Table 2. In terms of the mentioned properties of the porous structure, the parameters of zinc oxide and copper(II) oxide are very similar. The BET surface area for both ZnO and CuO is equal to 13 m^2^/g, which is four times lower than that of titanium(IV) oxide. As for the total volume of pores, for titanium(IV) oxide, it is at 0.020 cm^3^/g, while for the other two oxides it is at 0.005 cm^3^/g. The mean size of pores for all analyzed oxides is the same and amounts to 2.2 nm.

In the work of Zhang et al., titanium(IV) oxide was synthesized by hydrolysis of TiCl_4_ solution, and the obtained BET surface areas of TiO_2_ were equal to 35.9–289.9 m^2^/g, 5.0–167.6 m^2^/g and 20.5–271.4 m^2^/g for anatase, rutile and mixtures of both of these phases, respectively (the rutile weight fraction was between 0.58 and 0.85). The BET surface area of TiO_2_ is most developed when the lowest calcination temperature (110 °C) is used and it decreases with increasing calcination temperature (110–700 °C) [49]. Another study analyzed commercial titanium(IV) oxide, namely AEROXIDE^®^ TiO_2_ P25, which has a BET surface area (48 m^2^/g) only slightly less developed than the oxide analyzed in our study (53 m^2^/g) [50]. As for zinc oxide, in the work of Ahmad and others, nanometric ZnO particles synthesized from zinc acetate showed a more developed BET surface area (31.4 m^2^/g), compared to the commercial zinc oxide analyzed in our study (13 m^2^/g) [51]. In a further work, a zinc oxide precursor was prepared by precipitation from aqueous solutions of zinc nitrate and ammonium carbonate, and then the resulting intermediate was annealed to obtain nanometric ZnO. As a result of this process, it was possible to obtain particles with a BET surface area ranging from 25 to 125 m^2^/g. Here, the surface area was more developed when lower calcination temperature (250–450 °C) was used. This relationship was explained in such a way that a higher calcination temperature favors the formation of ZnO nanoparticles with larger diameters, as well as their aggregation [52]. In the work of Ren et al., the BET surface area of copper(II) oxide obtained with the use of thermal plasma technology (Tesima^TM^) was determined to be equal to 15.7 m^2^/g [53] and is comparable to the CuO surface area analyzed in our work (13 m^2^/g).

#### 3.1.5. Electrokinetic Properties

**Figure 4:**
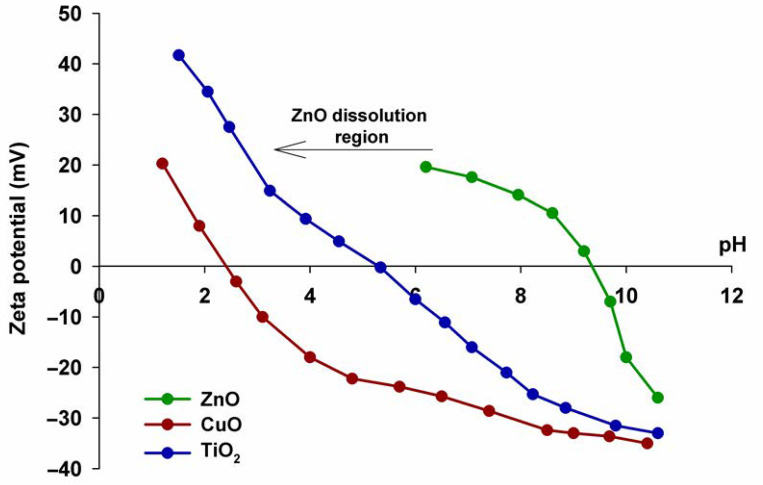
Zeta potential versus pH of pristine oxides.

In the next analysis, the electrokinetic potential was measured. The obtained zeta potential curves from the pH value for the tested pristine oxides are shown in Figure 4.

As can be seen in the case of titanium(IV) oxide, its particles possess a positive surface charge at low pH and, conversely, a negative surface charge at high pH. TiO_2_ thus exhibits relatively good electrokinetic stability (potential value ±20 mV) when the pH is lower than 3 and when its value exceeds 8, while the isoelectric point, being the intermediate pH at which the particle has a 0 surface charge, is equal to 5.3. This is a slightly lower value than in other works. For example, Jiang et al. observed that the isoelectric point for TiO_2_ was equal to 6 [54]. 

The work of Suttiponparnit et al. indicates that there are hydroxyl groups on the surface of nanoparticle titanium(IV) oxide when dispersed in water, which are formed according to the reaction (1):

(1) $${\mathrm{Ti}}^{\mathrm{IV}}+H_{2}O\to {\mathrm{Ti}}^{\mathrm{IV}}-\mathrm{OH}+H^{+}$$

The surface charge of titanium(IV) oxide, accordingly, depends on the pH of the solution, and the value of this parameter is influenced by the reactions occurring on the nanoparticle surface. This work also indicated that the increase in the surface charge of the particle may, as a result of increasing the electrostatic repulsive force, contribute to the inhibition of the agglomeration phenomenon and, consequently, to the reduction of the hydrodynamic size of the dispersion. In the further part of the work, it was shown that increasing the primary particle size of TiO_2_ from 6 to 104 nm reduces the isoelectric point from 6.0 to 3.8 [55].

In the case of copper(II) oxide (see Figure 4), the electrokinetic potential is higher in a more acidic environment. The isoelectric point for this oxide is equal to approximately 2.3 particles above this value, and it possesses a positive surface charge. Similar results were obtained in the work of El-Trass et al. [56], in which copper(II) oxide was synthesized in the reaction of copper acetate with sodium hydroxide in an ethanol environment. The obtained nanoparticle CuO exhibited positive zeta potential values at low pH and negative values at high pH, and, similar to the results presented in our work, the surface charge of copper(II) oxide decreased with increasing pH. The isoelectric point was observed at the pH value of 5.42, which is higher than that obtained in this Section 2.3.

The observed differences most likely result from the selection of different synthesis methods and, consequently, from the presence of various functional groups on the surface of CuO, e.g., such as the carboxyl groups mentioned in the cited work [56]. A report analyzing the influence of various shapes of copper(II) oxide nanoparticles, i.e., rice grain-like, needle-like and plate-like, on a number of CuO properties, including the zeta potential, can also be found in the literature. The obtained results allow a conclusion that CuO nanoparticles, regardless of their shape, show good stability in an aqueous environment, and the zeta potential values at pH = 7 are from −25 to −27 mV [57]. The study by Duman et al. also showed that CuO nanoparticles possess a negative surface charge of −20 mV [58].

As for zinc oxide, the isoelectric point for this compound is at a pH of 9.3. Below this pH, ZnO possesses a negative surface charge. Zinc oxide has relatively good electrokinetic stability at a pH below 10. Zinc oxide, like other metal oxides, has hydroxyl groups on its surface, which were also observed during the analysis of the FTIR spectrum of this oxide. Zn-OH layers are formed on the surface of the particles, which contribute to the generation of an appropriate surface charge. Thus, in neutral and alkaline environments, the protons absorbed by chemisorption pass into the solution, which causes the formation of a negative surface charge on which the partially bound Zn-O^−^ oxygen atoms are present. In a low pH environment, in contrast, protons are transferred to the surface, resulting in the formation of Zn-OH_2_^+^ groups on the surface, due to which zinc oxide has a positive surface charge. 

The work of Klapiszewska et al. indicates that zinc oxide has an isoelectric point at pH equal to 9.3. This is a relatively high value identical to the value obtained in this work. The cited report shows that such a high isoelectric point contributes to the fact that ZnO has a strong positive surface charge in neutral and acidic environments. Based on the analysis of the obtained curve, it was also found that ZnO forms electrokinetically stable dispersions only in an environment with a strongly basic pH [30]. The work of Berg et al. reveals that the isoelectric point for zinc oxide occurs at the pH value of 7.13; above this value, the charge present on the surface of the oxide is positive and is negative below it. Moreover, below pH 4 and above pH 8, ZnO nanoparticles form very electrokinetically stable dispersions (zeta potential value ±40 mV) [59].

Based on the analyses of the zeta potential versus pH curves, it can be concluded that all tested oxides exhibit good electrokinetic stability at high pH, which is of great importance for their potential use as admixtures for cement composites, due to their strongly alkaline environment.

### 3.2. Analysis of Cement Composites

The results of consistency, flexural strength and density tests for mortars with single zinc, copper(II) and titanium(IV) oxides and mixed systems of zinc oxide-copper oxide and titanium oxide-copper oxide at different weight ratios are presented in Table 3. Nanoadditives, due to their high fineness and BET surface areas, may limit the workability of cement mortars [2]. The study indicates that zinc oxide at 0.1 wt.%, copper(II) oxide at 0.5 wt.% and titanium(IV) oxide at 1 and 2 wt.% slightly improved the plasticity of mortars, while higher weight fractions of oxides used individually or in hybrid systems worsened this. For the flexural strength test, strength improvement was observed only for 0.1 wt.% zinc oxide and 1 wt.% titanium(IV) oxide, compared to pure mortar and the other oxide systems. Nevertheless, flexural strength and density increments, which illustrate the gradual hydration process of the cement binder, were observed for all mortars throughout the curing period.

**Figure 5:**
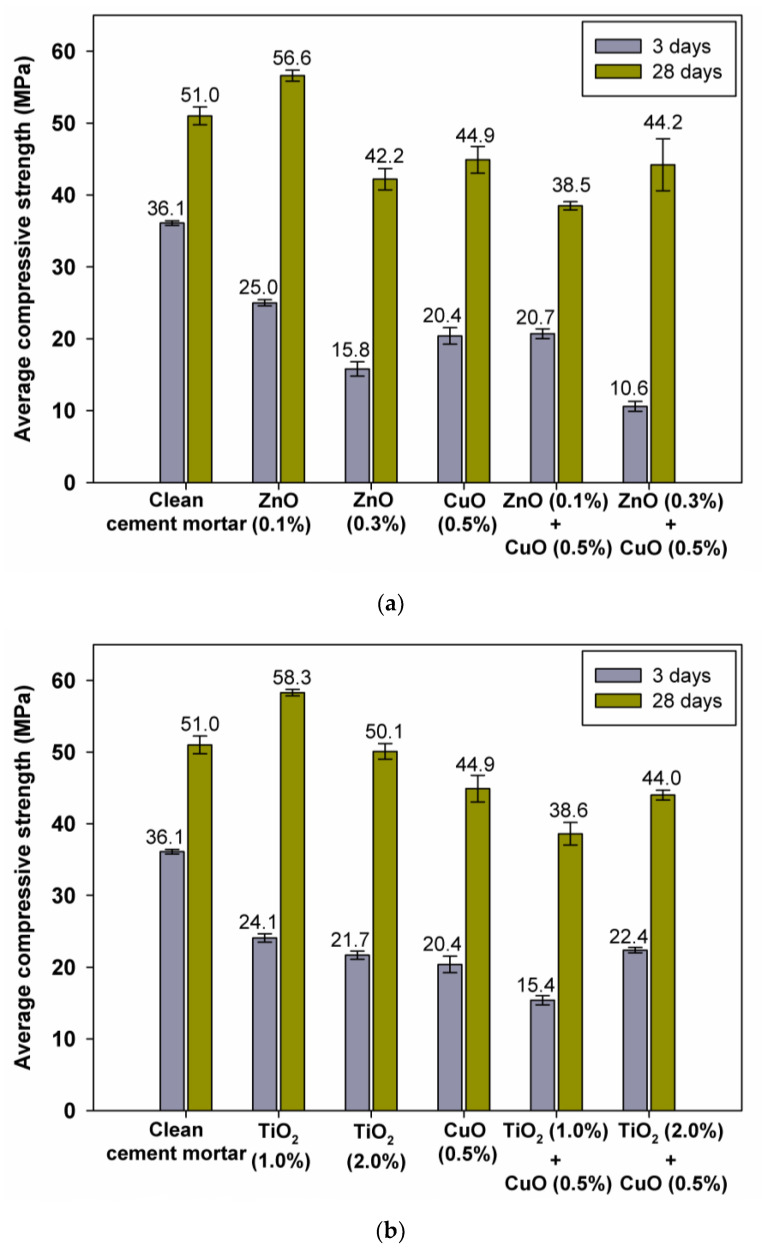
Average compressive strength of samples after 3 and 28 days (**a**,**b**).

The compressive strength results are presented in Figure 5. Based on the conducted research, some trends can be observed. During the initial period of cement matrix hardening, up to day 3, we observed that zinc oxide at both weight percentages and copper(II) oxide affected the setting retardation, as manifested by lower strength gains compared to pure mortar. Similar relationships have also been reported for titanium(IV) oxide, with higher strength decreases. Despite the retarding effect of all oxides on cement binder setting during the first few days, an improvement in compressive strength after 28 days of curing was observed for both zinc and titanium(IV) oxides used in the amounts of 0.1 wt.% and 1 wt.%, respectively. This shows that these levels of oxides are the optimal values to guarantee strength gains throughout the test period.

According to the theory of nanoadditives action, in this case, the applied oxides may constitute additional active centers from which the nucleation and growth of the C-S-H phase starts. This results in the thickening of the structure and acceleration of the hydration process and manifests itself in the increase of strength, as compared to pure mortar [2,5,6]. 

**Figure 6:**
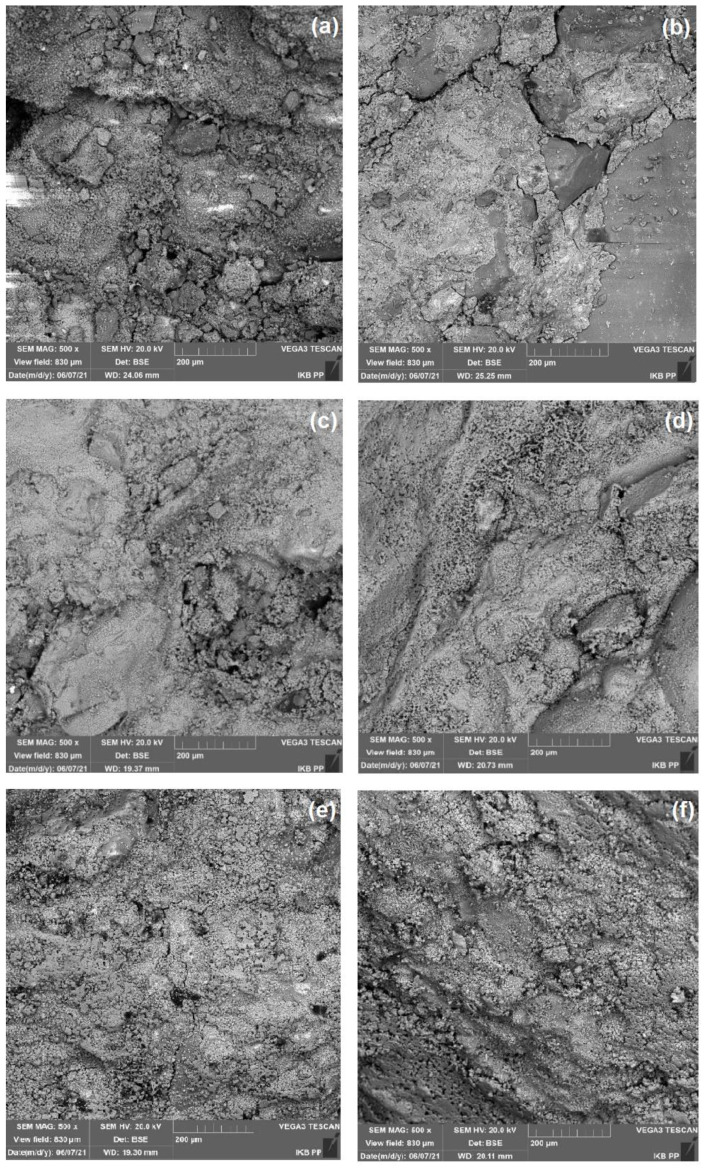
SEM images of clean cement mortar (**a**) and cement composites with ZnO (0.1%) (**b**); CuO (0.5%) (**c**); TiO_2_ (1.0%) (**d**); ZnO (0.1%) + CuO (0.5%) (**e**); TiO_2_ (1.0%) + CuO (0.5%) (**f**).

Higher contents of both nanoadditives, however, induced a significant deterioration of strength parameters. In the case of copper(II) oxide, a decrease of mechanical parameters was observed throughout the study period, both for copper(II) oxide used alone and in a system with zinc and titanium(IV) oxide. This may indirectly result from the deterioration of the workability of cement mortars, which was evident in the significant deterioration of their plasticity, especially for samples with higher amounts of oxides. This is reflected in the microstructure analysis of the cement mortars shown in Figure 6. In the case of cement mortars with zinc oxide and copper(II) oxide (shown in Figure 6b,c, respectively), a compact and well-densified cement matrix microstructure and good adhesion at the cement paste-aggregate interface can be observed. In the case of titanium(IV) oxide, for which much higher amounts of nanoadditive were used, a loosening of the structure was observed, which is further aggravated when double oxide systems are used. 

For the mortar shown in Figure 6e,f, an increase in the porosity of the cement paste microstructure is notable, as well as numerous free spaces that remained after water evaporation. These effects may also result from the uneven mixing of mortar components due to limited workability with higher amounts of nanoadditives. 

In our work, we saw that the increased flexural strength with 0.1 wt.% zinc oxide results from better adhesion of the cement paste to the aggregate. We also noted that better compressive strength, better than both copper(II) oxide and zinc oxide, was shown for titanium(IV) oxide at 1 wt.%. Similar effects of titanium oxide addition on mechanical properties have been observed by other researchers. For example, Lucas et al. showed that anatase in the P25 variety added to cement-lime mortars in amounts ranging from 0.5, 1, 2.5, and 5 wt.%, in amounts up to 1 wt.% resulted in higher compressive strength values but lower flexural strength values compared to mortars without the additive [60]. 

Different behaviors of titanium oxide in compressive strength tests depending on the amount of additive were also observed by Ma et al. [61]. Therein, titanium oxide added to cement mortars and concretes in amounts up to 3 wt.% induced an increase in flexural strength, while higher amounts brought about a decrease in both flexural and tensile strength. On the other hand, in many works, titanium oxide dosed both in lower amounts up to 5 wt.% as well as higher, 5 to 10 wt.%, resulted in more or less improvement in compressive strength, with significantly higher strength gains observed during the initial curing period of the cementitious composites [62,63,64,65,66].

This outcome is thought to be due to the higher ratio of the oxide in the cement matrix, which contributes to a significant increase in the number of active centers compared to the other oxides, from which the growth of the C-S-H phase and the thickening of the structure starts. In our work, the decrease in strength at 2 wt.% is probably due to amounts of the nanoadditive used that were too high, which contributes to worse workability of the mortar and worse dispersion of titanium(IV) oxide in the cement matrix, and consequently to a deterioration of both flexural and compressive strengths. This causes titanium(IV) oxide to agglomerate at higher weight percentages, thus losing some of its properties. 

Although the particle distributions of titanium(IV) oxide and zinc oxide are comparable, the specific surface area and applied amounts of titanium(IV) oxide are much higher. This makes titanium oxide significantly affect the rheological and mechanical properties of the cement matrix. Therefore, with such a selected composite composition, the optimum amount for titanium(IV) oxide is 1 wt.%. In the case of the combination of titanium(IV) oxide and zinc oxide with copper(II) oxide, copper(II) oxide is responsible for a significant decrease in strength parameters, which notably delays the setting of the cement binder and contributes to a decrease in the mechanical parameters of the cement composite.

### 3.3. Assessment of Antibacterial Properties

**Figure 7:**
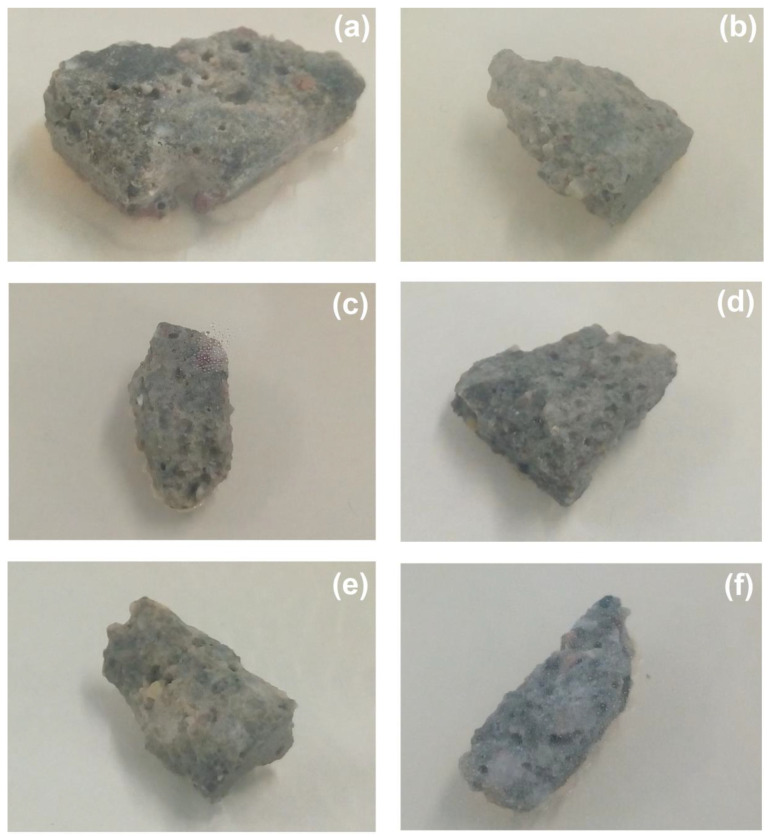
Example images of samples subjected to microbial purity testing: (**a**) clean cement mortar; (**b**) ZnO (0.1%); (**c**) ZnO (0.3%); (**d**) CuO (0.5%); (**e**) TiO_2_ (1.0%); (**f**) ZnO (0.1%) + CuO (0.5%).

The first stage of assessment of the antimicrobial properties was focused on a relatively simple and rapid evaluation of microbial purity. This test allows for visual inspection of microbial growth in the direct vicinity of the studied sample and indicates possible antimicrobial activity at the agar/sample interface. The results are summarized in Table 4, while example images that visualize the microbial purity of selected samples are presented in Figure 7.

Overall, the use of the studied oxide admixtures improved the microbial purity of the samples. The highest microbial growth was observed in the case of clean cement mortar. As presented in Figure 7a, extensive microbial growth was visible on each side of the cement sample and the biomass occurred in the form of multiple colonies. In contrast, not even a single colony appeared in the case of cement samples supplemented with ZnO (0.1%), CuO (0.5%) or TiO_2_ (1.0%) (Figure 7b,d,e, respectively). This indicates that the presence of the oxides contributed to an inhibitory effect. Interestingly, application of higher content of the oxides (e.g., ZnO (0.3%) or TiO_2_ (2.0%)) resulted in slight microbial growth (the growth of a few colonies was visible under the sample, as presented in Figure 7c). A similar effect occurred when a mixture of oxides was used (barely visible for ZnO (0.1%) + CuO (0.5%) in Figure 7f). 

In the second part of the study, the characteristic antimicrobial parameters (MIC and MBC/MFC) were determined. The employed test allows evaluating the antimicrobial properties of the tested compounds towards selected strains of bacteria and fungi. The results are presented in Table 5.

A comparison of the obtained results indicates that the antimicrobial effect depends on both the studied material and the tested microorganism. The samples with CuO (0.5%) displayed the highest activity against the tested strains. The lowest MIC and MBC values (equal to 0.05 and 0.25 g/L, respectively), which correspond to the highest antimicrobial activity, were observed for *E. coli*, whereas the highest (MIC = 1.25 g/L; MBC = 5 g/L) were noted for *S. aureus*. Among the two remaining oxides, ZnO (0.1%) showed higher efficiency against *S. aureus* and *E. coli*, while TiO_2_ (1.0%) inhibited the growth of *B. cereus* and *P. aeruginosa* to a greater extent. The worst results were observed in the case of clean cement mortar, which did not inhibit the growth of the studied microorganisms in the tested range of concentrations. 

The susceptibility of the studied strains towards the samples also differed notably. In the case of bacteria, there was no visible trend regarding the Gram-negative and Gram-negative species. Based on the inhibitory effect of the studied compounds, the following order of microorganisms can be established (from the most susceptible to the most resistant): *E. coli* < *S. aureus* < *C. albicans* < *B. cereus* = *P. aeruginosa* < *P. putida*.

The antimicrobial properties of various inorganic oxides have been the object of numerous studies [67,68]. There are several possible advantages resulting from the incorporation of such materials into products of everyday use, a few examples of which include extended shelf-life of food products [69], higher dental hygiene [70] or improved safety against harmful pathogens [71]. Self-cleaning cement with an admixture of active oxides is a perfect example of the last application area. Such materials can be of high importance in areas susceptible to the formation of biofilms by pathogenic bacteria (e.g., hospitals). 

The results obtained in this study confirm that the oxide-supplemented cement samples exhibited antimicrobial properties; however, the exact effect depended on (i) the type of oxide; (ii) its content in the sample and (iii) the tested microbial species. We established that cement samples with CuO (0.5%) exhibited the most inhibitory effect. Higher antimicrobial activity of CuO compared to ZnO against *S. aureus*, *E. coli* and *P. aeruginosa* was also observed by Dadi et al. [72].

While the benefits of employing oxide materials are tempting, it should also be pointed out that, depending on their application, some risks are involved (e.g., toxicity against nonintended organisms) [73,74]. Furthermore, there are no standards regarding the study of antimicrobial properties of such materials, which corresponds to several inconsistencies in the current state-of-the-art. The lack of uniform testing procedures results in ambiguity of data from different reports [75]. Additionally, the method of preparation of the oxide nanoparticles may also significantly affect their mechanism of action and thus impact their activity [30]. Hence, there is a need to employ tests that allow for numeric determination of antimicrobial effects against numerous species and to combine them with appropriate physicochemical characterization of the studied materials in order to allow for their clear comparison. We believe that the approach presented in this study, which provides an in-depth analysis of the materials characteristics and a two-step investigation of their antimicrobial properties (visual examination combined with determination of MIC, MBC and MFC) is the bare minimum necessary for filling the data gaps.

## 4. Conclusions

This work has been divided into two main parts. The first analyzes a number of properties of zinc oxide, copper(II) oxide and titanium(IV) oxide. The absence of additional bands in the FTIR spectra as well as the peaks in the EDXRF spectra of the tested oxides confirms that the correct oxides were used. These oxides are contaminated to a small extent with residues from the production process (ZnO and CuO) and contain hydroxyl groups (TiO_2_ and ZnO) on their surfaces. The particle size of ZnO, CuO and TiO_2_ is nanometric, and the TiO_2_ particles are the least homogeneous in terms of size. The most developed BET surface area is that of titanium(IV) oxide (53 m^2^/g), which is four times greater than that of the other two oxides. A similar dependence can be observed for the total volume of pores, which is equal to 0.020 cm^3^/g for TiO_2_ and 0.005 cm^3^/g for ZnO and CuO. All analyzed oxides exhibit relatively good electrokinetic stability (zeta potential below −20 mV) in an alkaline environment, which is important for their use in cement composites with a strongly alkaline environment.

In the second part of the study, we determined the dosage range of oxides used individually and in binary systems, and the effect of oxides on the basic physicomechanical properties of cement mortars. In addition, their inhibition ability towards selected bacteria and fungi was investigated. Our work indicates that during the initial period of mortar hardening, all oxides caused a significant deterioration of the strength parameters, both flexural and compressive. Nevertheless, gradual increases in strength were observed in the long term. Indeed, higher compressive strengths were recorded for zinc and titanium(IV) oxide at 0.1 and 1 wt.% after 28 days of curing than for pure mortar and other oxide systems. A significant deterioration of strength parameters was observed for copper(II) oxide and its combination with zinc and titanium(IV) oxides, which in the case of binary systems was indirectly associated with deterioration of the plasticity and workability of mortars. 

Among all the tested nanoadditives, copper(II) oxide exhibited the highest ability to inhibit the growth of bacteria and fungi. The study further demonstrated the selective activity of the nanoadditives towards Gram-positive and Gram-negative pathogens. 

The work also revealed that the amount of nanoadditives and their proper dispersion in the cement matrix is an important factor. The use of single oxides proved to be a much better solution than the use of binary oxide systems, wherein deterioration of workability and strength parameters of the cement matrix were observed. This could result in an uneven distribution of the nanoadditives in the mortars and nullification or even inhibition of their antibacterial activity.

## Figures and Tables

**Table 1:** The composition of the mixtures used to prepare the appropriate cement composites.

Sample Name	Quantities of Ingredients Used in the Preparation of Cement Composites					
	Cement [g]	Water [mL]	Aggregate [g]	ZnO [g]	CuO [g]	TiO_2_ [g]
clean mortar cement	450	225	1350	-	-	-
ZnO (0.1%)				0.45	-	-
ZnO (0.3%)				1.35	-	-
CuO (0.5%)				-	2.25	-
TiO_2_ (1.0%)				-	-	4.50
TiO_2_ (2.0%)				-	-	9.00
ZnO (0.1%) + CuO (0.5%)				0.45	2.25	-
ZnO (0.3%) + CuO (0.5%)				1.35	2.25	-
TiO_2_ (1.0%) + CuO (0.5%)				-	2.25	4.50
TiO_2_ (2.0%) + CuO (0.5%)				-	2.25	9.00

**Table 2:** Tabulated data of dispersive properties and porous structure properties of pristine oxides.

Sample	Dispersive Properties		Porous Structure		
	Particle Size Distribution from Zetasizer Nano ZS (nm)	PdI	BET Surface Area (m^2^/g)	Total Volume of Pores (cm^3^/g)	Mean Size of Pores (nm)
ZnO	142–712	0.139	13	0.005	2.2
CuO	220–1484	0.140	13	0.005	2.2
TiO_2_	190–396	0.788	53	0.020	2.2

**Table 3:** Consistency, flexural strength and density of cement mortars containing oxides in varying amounts.

Sample	Consistency (mm)	Flexural Strength (MPa)		Density (g/cm^3^)	
		After 3 Days	After 28 Days	After 3 Days	After 28 Days
clean cement mortar	135.0 ± 5.0	4.5 ± 0.4	7.0 ± 0.1	2.31 ± 0.02	2.36 ± 0.01
ZnO (0.1%)	134.3 ± 5.8	4.7 ± 0.1	7.7 ± 0.4	2.28 ± 0.01	2.32 ± 0.01
ZnO (0.3%)	127.8 ± 3.7	2.9 ± 0.2	6.8 ± 0.1	2.20 ± 0.01	2.23 ± 0.01
CuO (0.5%)	138.5 ± 3.0	4.2 ± 0.4	6.6 ± 0.1	2.28 ± 0.02	2.28 ± 0.02
TiO_2_ (1.0%)	146.5 ± 1.7	4.8 ± 0.1	8.5 ± 0.2	2.28 ± 0.01	2.39 ± 0.02
TiO_2_ (2.0%)	136.0 ± 4.0	4.4 ± 0.2	7.2 ± 0.2	2.31 ± 0.03	2.36 ± 0.04
ZnO (0.1%) + CuO (0.5%)	126.8 ± 1.9	4.1 ± 0.1	6.6 ± 0.4	2.22 ± 0.02	2.28 ± 0.03
ZnO (0.3%) + CuO (0.5%)	131.0 ± 10.7	2.6 ± 0.1	7.3 ± 0.1	2.20 ± 0.01	2.25 ± 0.03
TiO_2_ (1.0%) + CuO (0.5%)	132.8 ± 6.2	3.2 ± 0.2	6.1 ± 0.4	2.26 ± 0.03	2.32 ± 0.03
TiO_2_ (2.0%) + CuO (0.5%)	134.5 ± 3.0	4.4 ± 0.4	7.3 ± 0.1	2.37 ± 0.03	2.29 ± 0.03

**Table 4:** Results of microbial purity testing for all materials.

Sample	Microbial Purity (After 24 h)	Comments
clean cement mortar	+++	Extensive microbial growth (>2 mm) on each side, numerous colonies visible under the sample
ZnO (0.1%)	−	No microbial growth
ZnO (0.3%)	−/+	Few colonies visible under the sample
CuO (0.5%)	−	No microbial growth
TiO_2_ (1.0%)	−	No microbial growth
TiO_2_ (2.0%)	−/+	Few colonies visible under the sample
ZnO (0.1%) + CuO (0.5%)	−/+	Few colonies visible under the sample
ZnO (0.3%) + CuO (0.5%)	−/+	Few colonies visible under the sample
TiO_2_ (1.0%) + CuO (0.5%)	−/+	Few colonies visible under the sample
TiO_2_ (2.0%) + CuO (0.5%)	−/+	Few colonies visible under the sample

“+” indicates microbial growth, whereas “−” indicates a lack of thereof.

**Table 5:** Determination of antimicrobial properties of the studied materials (expressed as MIC and MBC/MFC).

Sample	*Bacillus* *cereus*		*Staphylococcus aureus*		*Pseudomonas aeruginosa*		*Pseudomonas putida*		*Escherichia coli*		*Candida albicans*	
	MIC [g/L]	MBC [g/L]	MIC [g/L]	MBC [g/L]	MIC [g/L]	MBC [g/L]	MIC [g/L]	MBC [g/L]	MIC [g/L]	MBC [g/L]	MIC [g/L]	MFC [g/L]
clean cement mortar	above 20	above 20	above 20	above 20	above 20	above 20	above 20	above 20	above 20	above 20	above 20	above 20
ZnO	above 20	above 20	5	20	above 20	above 20	above 20	above 20	0.05	1.25	5	20
CuO	0.05	1.25	1.25	5	0.05	1.25	0.25	1.25	0.05	0.25	0.05	1.25
TiO_2_	10	above 20	20	above 20	10	above 20	20	above 20	1.25	5	5	above 20
